# Intra-Arterial Chemotherapy for Advanced Intraocular Retinoblastoma…

**DOI:** 10.1007/s00270-026-04434-2

**Published:** 2026-04-15

**Authors:** Sena Bozer Uludağ, Emre Can Çelebioğlu, Emre Utkan Büyükceran, Didenur Karnak, Mehmet Yılmaz, Fatmanur Duruk Erkent, Seda Kaynak Şahap, Süheyla Karadağ Erkoç, Ahmet Kaan Gündüz, Sadık Bilgiç

**Affiliations:** 1 Department of Radiology, Tosya State Hospital, Kastamonu, Turkey; 2 Department of Radiology/Vascular Interventional Radiology, School of Medicine, Ankara University Faculty of Medicine, Ankara, 06100 Turkey; 3 Department of Radiology, Güven Hospital, Ankara, Turkey; 4 Department of Ophthalmology, School of Medicine, Ankara University, Ankara, Turkey; 5 Department of Anatomy, Ankara University Faculty of Medicine, Ankara, Turkey; 6 Department of Anesthesiology and Reanimation, School of Medicine, Ankara University, Ankara, Turkey; 7 Department of Radiology/Pediatric Radiology, School of Medicine, Ankara University, Ankara, Turkey

**Keywords:** Retinoblastoma, Intra-arterial chemotherapy, Ophthalmic artery, Pediatric oncology, Eye preservation

## Abstract

**Purpose:**

To evaluate the safety and efficacy of intra-arterial chemotherapy as a treatment for advanced intraocular retinoblastoma and to present our single-center experience.

**Materials and Methods:**

This single-center retrospective cohort study included 13 pediatric patients (15 eyes) who underwent intra-arterial chemotherapy between January 2020 and December 2024. Patients were categorized into two groups: those receiving treatment as a first-line option (group 1) and those treated following the failure of previous therapies (group 2). Data were collected on technical success, complications, ocular preservation, and visual function. Each patient was monitored for a minimum of 12 months after treatment initiation.

**Results:**

According to the ICRB classification, most eyes (66.7%) were Groups D, followed by groups C and E. The mean age at presentation was 22.5 months. A total of 45 intra-arterial chemotherapy procedures were performed, with successful catheterization achieved in 97.8% of cases. Minor procedural complications were observed in 8.8% of sessions, with no serious systemic adverse events. Visual acuity was maintained or improved in 93.3% of treated eyes. No patients required enucleation, and no cases of metastasis or death occurred during follow-up.

**Conclusion:**

Intra-arterial chemotherapy is a technically feasible and promising globe-sparing treatment for intraocular retinoblastoma, demonstrating high technical success and a favorable safety profile in our cohort. While preliminary outcomes show preserved visual function and high ocular survival, larger studies with longer follow-up are necessary to confirm long-term efficacy and safety within a multidisciplinary framework.

**Graphical Abstract:**

**Figure:**
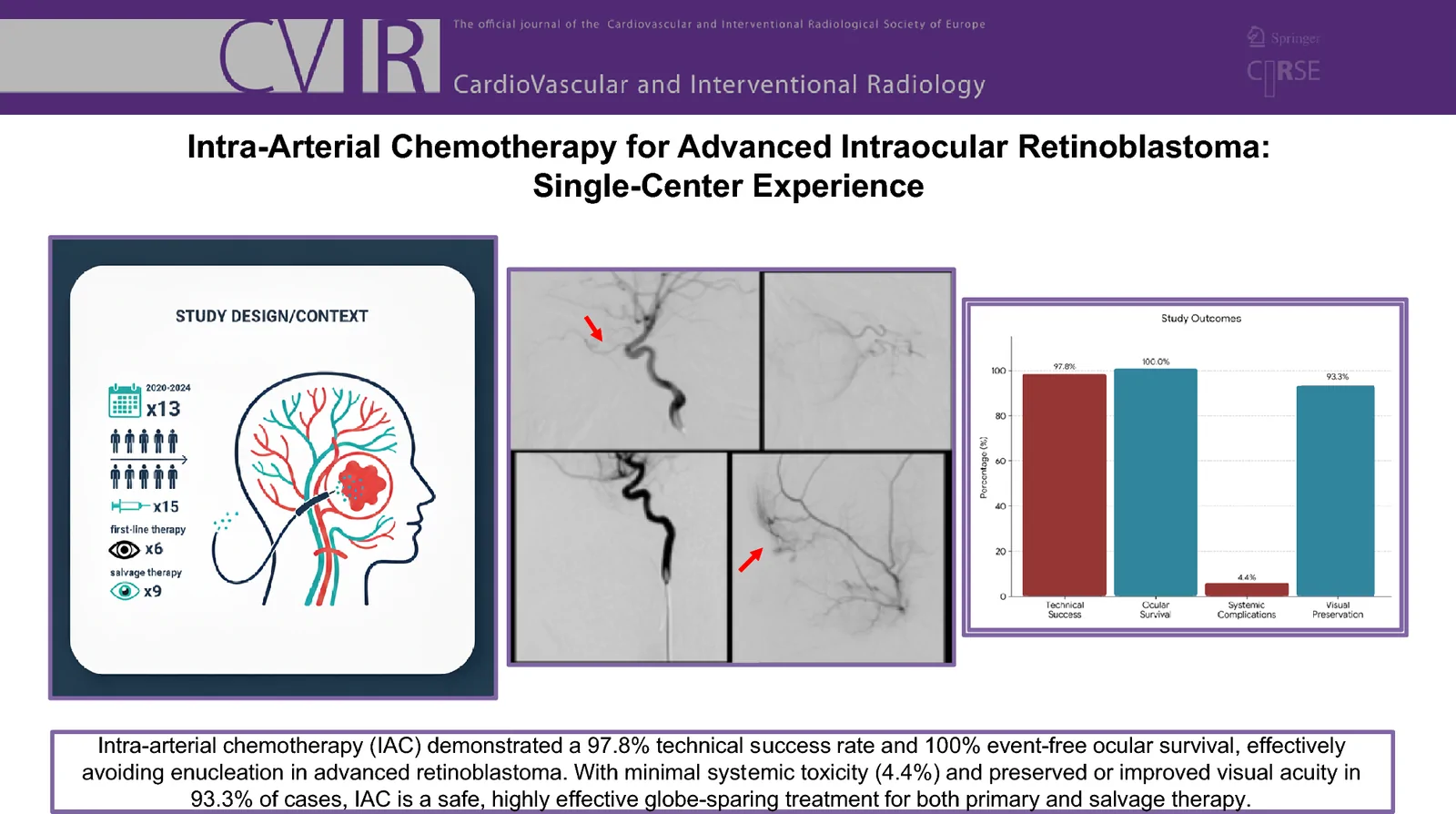

## Introduction

Retinoblastoma (RB) is the most common primary intraocular malignancy in childhood, typically diagnosed before age five [1, 2]. Prognosis is strongly stage-dependent [3, 4]. Although fatal if untreated, early detection and appropriate therapy can achieve cure rates exceeding 90% in developed countries [2, 5]. Historically, enucleation was the mainstay treatment, especially in resource-limited settings [6, 7].

Today, multiple eye-preserving therapies are available, including local treatments, radiotherapy, intraocular chemotherapy [intravitreal chemotherapy (IvitC), intracameral chemotherapy (IcamC)], intravenous chemotherapy (IVC), and intra-arterial chemotherapy (IAC) [2]. While practices vary, there is a global shift toward globe-sparing strategies aiming to balance life preservation, metastasis prevention, and visual function [2, 3, 8]. 

IAC, introduced in the 1950s, gained traction after technical advances in superselective ophthalmic artery (OA) catheterization [7, 9, 10]. It allows high intraocular drug concentrations with reduced systemic toxicity compared to IVC [2, 8, 11]. Although Melphalan is the agent of choice due to its potent intraocular activity, triple-drug combinations including carboplatin and topotecan have also been adopted [7, 11–14]. Recent comparative evidence from Alshahrani et al. has demonstrated that triple-agent IAC regimens are associated with improved tumor response and ocular survival, while maintaining a manageable safety profile with predominantly transient complications [14].

IAC serves as both first-line therapy (for unilateral, non-germline group B–E disease) and salvage therapy in recurrent or seed-rich cases [2, 11]. Particularly in group D/E tumors, IAC outperforms IVC in globe salvage [11, 15, 16]. Despite the expanding literature, evidence remains limited regarding standardized triple-agent IAC protocols applied in real-world settings without balloon-assisted techniques, and interventional radiology–focused procedural outcomes and complication grading are underreported. The aim of this study was to evaluate the technical success, safety, and clinical outcomes of a standardized triple-agent IAC regimen, performed without balloon assistance, as a globe-sparing strategy for intraocular retinoblastoma, based on our single-center experience.

## Materials and Methods

This single-center retrospective cohort study was conducted at a tertiary referral center and included patients who underwent IAC for RB between 2020 and 2024. The study was approved by the Institutional Research Ethics Committee of XXX University (Approval No: 2025000191, dated March 10, 2025). Written informed consent was obtained from the legal guardians of all patients, and all procedures were performed in accordance with the 1964 Helsinki Declaration and its later amendments. Patient data were anonymized before analysis.

Patients were included in this study if they met the following criteria: (1) a confirmed diagnosis of intraocular RB made by a multidisciplinary team, (2) treatment with at least one IAC session at our institution between January 2020 and December 2024, and (3) available complete clinical and imaging follow-up data. Exclusion criteria were the evidence of extraocular extension or systemic metastasis at the time of diagnosis.

Data collection followed the Global Retinoblastoma Study Group parameters. Tumors were staged using the 8th edition AJCC classification and grouped by the International Classification of Retinoblastoma (ICRB). Eyes were divided into two groups based on their treatment history: Group 1 received IAC as first-line therapy, which is the initial treatment modality for treatment-naive cases. Group 2 received IAC as salvage therapy, defined as the administration of IAC following the failure or progression of prior therapies, including IVC, IvitC, or local treatments. Multidisciplinary team decisions guided the use of IAC.

Data on catheterization success and complications were recorded. The minimum follow-up was 12 months. Primary outcomes included procedural success, complications, event-free ocular survival (without enucleation), metastasis, and mortality. Technical success was defined as successful superselective OA catheterization and complete delivery of the planned chemotherapeutic dose. Adverse events were classified and reported according to the CIRSE classification system.

### IAC Procedure and Treatment Protocol

IAC was performed by two interventional radiologists with 10 (E.C.Ç) and 40 (S.B) years of experience using a biplane angiography system (Artis Zee, Siemens, Germany). Under general anesthesia, the femoral artery was accessed via ultrasound guidance (Sonosite, USA) with a 4F sheath (Terumo, Japan). Anticoagulation was achieved with a single intravenous bolus of unfractionated heparin at a dose of 50 IU/kg. Routine activated clotting time monitoring was not performed, consistent with commonly reported pediatric intra-arterial chemotherapy protocols, given the relatively short procedural duration and the superselective nature of ophthalmic artery catheterization. Additional heparin boluses were not routinely administered. A 4F vertebral catheter (Terumo, Japan) was advanced into the intended carotid artery, and a microcatheter (Marathon, Medtronic, USA; Headway Duo, MicroVention, USA or Echelon, Medtronic, USA) with a compatible guidewire (e.g.; Synchro, Stryker, USA; Asahi Chikai, Asahi Intecc, Japan; Mirage, Medtronic, USA) were used to navigate into the OA via the internal carotid artery (ICA) or external carotid artery (ECA) depending on OA origin.

**Fig. 1:**
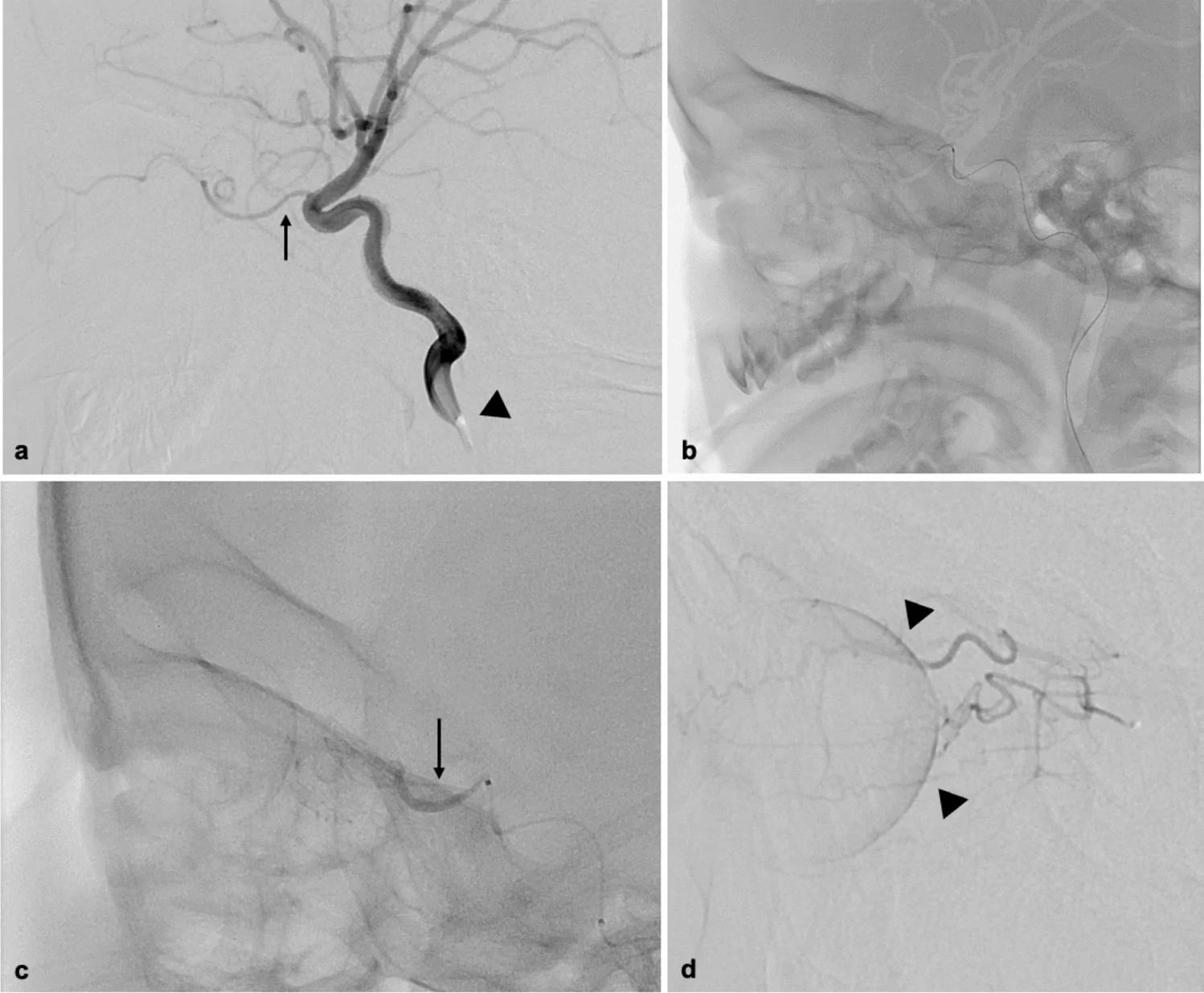
Preferred IAC technique for a right-sided unilateral retinoblastoma. **a** Lateral subtracted angiogram shows a 4-French guide catheter (*arrowhead*) in the distal internal carotid artery and the ophthalmic artery (*arrow*). **b** Lateral unsubtracted view after superselective catheterization of the ophthalmic artery. **c**-**d** Unsubtracted and subtracted angiograms show patency of the ophthalmic artery (*arrow*) and a choroidal blush (*arrowheads*)

**Fig. 2:**
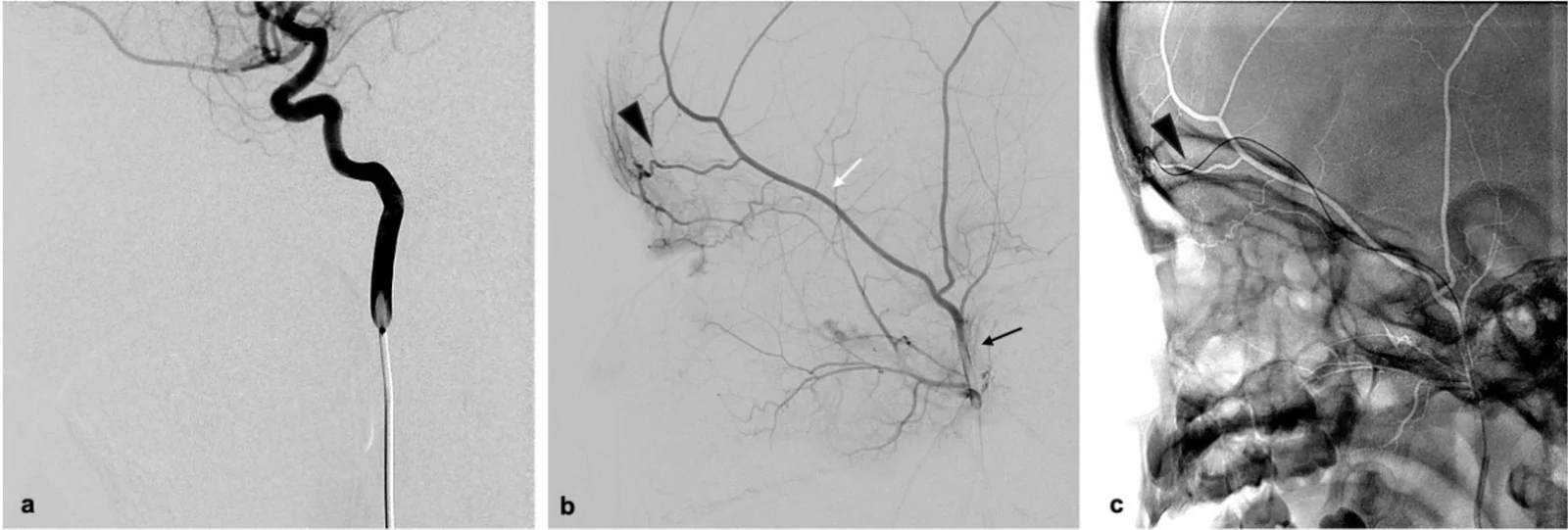
Meningo-ophthalmic variant used for an alternative IAC technique. **a** lateral subtracted angiogram following internal carotid artery injection demonstrates absence of choroidal blush. **b** lateral subtracted angiogram shows a 4-French guide catheter (*black arrow*) in the distal external carotid artery, the middle meningeal artery (*white arrow*), and the meningo-ophthalmic artery (*arrowhead*). **c** lateral roadmap image demonstrates superselective catheterization of the meningo-ophthalmic artery with a microguidewire (*arrowhead*)

Superselective angiography confirmed OA cannulation and choroidal blush (Fig. 1). If necessary, access was obtained via the middle meningeal artery (MMA), or the related branch of the ECA (Fig. 2). The Japanese technique using ICA balloon occlusion was not utilized [17].

Chemotherapy was manually infused at 1 mL/min over 30 min using a pulsatile technique under fluoroscopy. Total volume was 30 mL with intermittent flushing to prevent vasospasm. A final control angiogram excluded thromboembolism. Hemostasis was achieved via manual compression. Patients were discharged the same day after 4–6 h of observation. CBCs were obtained 7–10 days post-IAC [7, 11].

Treatment intervals were 3–4 weeks. Each session was preceded by ophthalmologic evaluation under anesthesia. Though a 3-cycle regimen is standard [7, 11], the number of sessions was individualized based on tumor response.

The chemotherapy protocol included *Melphalan* (*Melphalan Tillomed®*, Tillomed Pharma GmbH, Germany), *Topotecan* (*Topotu®*, Koçak Farma, Türkiye), and *Carboplatin* (*Carboplatin-Koçak®*, Koçak Farma, Türkiye). Melphalan dosage was age-based: 2.5 mg (3–6 months), 3 mg (6–12 months), 4 mg (1–3 years), and 5 mg (> 3 years) [7, 9]. Topotecan was given as 0.3 mg (< 3 years) or 0.4 mg (≥ 3 years). Carboplatin (30 mg) was used only in patients aged ≥ 6 months. For bilateral treatments, body weight guided the total dose [7]. In cases of same-day bilateral intra-arterial chemotherapy, the cumulative dose calculated according to body weight was distributed between both eyes to remain within systemic dose limits. Although a standard three-cycle IAC regimen was generally intended, the total number of cycles was individualized based on multidisciplinary tumor board decisions and treatment response.

All analyses were performed using SPSS v20. Categorical variables were reported as frequencies and percentages; continuous variables were expressed as means ± SD or medians (IQR), depending on distribution. Normality was assessed using the Shapiro–Wilk test. In addition to descriptive statistics, 95% confidence intervals (CIs) for key proportions were calculated using the Clopper–Pearson exact method. To analyze visual acuity across two different methods, results were converted into a standardized ordinal scale (improved, stable, or worsened). Differences in these categorical outcomes between groups were evaluated using the Fisher–Freeman-Halton exact test. For continuous variables the Mann–Whitney U test was employed to compare the medians between groups. Significance was set at *p* ≤ 0.05.

## Results

Thirteen patients (15 eyes, 7 males and 6 females) were included. The mean age at presentation was 22.5 months (range, 4–85 months). Most eyes were ICRB group D (66.7%), followed by groups C, E (13.3%), and B (6.7%). Seven had unilateral, and six had bilateral disease; IAC was delivered bilaterally in two and unilaterally in four patients. None had trilateral disease, a family history, or an RB1 mutation. The mean age at first symptom was 19.8 months (range, 1–90). The most common signs were leukocoria (*n* = 5) and strabismus (*n* = 5), followed by nystagmus (*n* = 2) and decreased vision (*n* = 1) (Table 1).

**Table 1:** Demographic and clinical characteristics of patients

	*n* =	%
Patient	13	
Eye	15	
Male	7	53.8%
Age (months)^a^		
At the onset of the first symptom	19.8 ± 24.2	
minimum-maximum	*1–90*	
At the time of the initial presentation	22.5 ± 22.4	
minimum-maximum	*4–85*	
First Symptoms		
Leukocoria	5	38.5%
Squint	5	38.5%
Nystagmus	2	15.3%
Visual acuity deterioration	1	7.7%
Family History	0	0%
Heritable Trait		
No evidence of RB1 mutation	13	100%
Laterality		
Unilateral	7	53.9%
Right	*5*	*71.4%*
Left	*2*	*28.6%*
Bilateral	6	46.1%
Trilateral	0	0%
AJCC TNM Stage		
I	14	92.3%
II	0	0%
III	1	7.7%
IV	0	0%
ICRB Group		
A	0	0%
B	1	6.7%
C	2	13.3%
D	10	66.7%
E	2	13.3%

^a^Mean ± Standard deviation

AJCC TNM: Tumor (T), lymph node (N) and metastasis (M) status according to the 8th edition of the American Joint Committee on Cancer (AJCC) classification; ICRB International Classification of Retinoblastoma, L left eye, R right eye, RB1 The retinoblastoma protein gene

IAC was first-line treatment in six eyes (Group 1) and salvage therapy in nine (Group 2). Overall, 45 procedures were performed, with a median of 4 cycles per eye (range, 1–7). The intensity of treatment differed significantly between the two groups (*p* = 0.005). Patients in Group 2 required a higher number of treatment cycles compared to Group 1 (Table 1). 

In four patients, catheterization required an ECA access. Successful OA catheterization was achieved in 44 of 45 attempts (97.8%; 95% CI 88.2–99.9%). One failure due to vasospasm was corrected at repeat procedure (grade 1a complication; according to the Modified CIRSE Classification System) [18]. Our analysis revealed that the anatomical origin of the OA did not significantly influence the catheterization success or the incidence of post-procedural complications (*p* = 1.000).

Intra-procedural complications occurred in four sessions (8.8%; 95% CI 2.5–21.2%), all of which were procedure-related. These included transient bronchospasm in three sessions (6.6%) and hypotension in one session (2.2%), all classified as Grade 3a complications according to the Modified CIRSE Classification System [18]. Post-procedural complications were observed following six sessions (13.2%; 95% CI 5.1–26.8%). Drug-related ocular events consisted of periocular inflammation in four sessions (8.8%; Grade 2), while systemic events occurred in two sessions, presenting as self-limited rash and pancytopenia (4.4%; Grade 2) [18] (Table 2).

**Table 2:** Procedural details of IAC

	*n* =	%	95% CI
IAC cycle^a^	4 (2–4)		
minimum–maximum	1–7		
Group 1	2 (1.75–4.5)		
minimum–maximum	1–10		
Group 2	6 (6–7.5)		
minimum–maximum	6–11		
IAC catheterization	45		
Unilateral	39	86.7%	
Bilateral	6	13.3%	
Catheterization success	44	97.8%	88.2–99.9%
Procedure-related complication	*1*	*2.2%*	
Vasospasm*	*1*	*2.2%*	
Intra-procedural complications	*4*	*8.8%*	*2.5–21.2%*
Procedure-related	4	8.8%	
Transient bronchospasm**	3	6.6%	
Hypotension**	1	2.2%	
Drug-related	*0*	*0%*	
Post-procedural complications	6	13.2%	5.1–26.8%
Procedure-related	*0*	*0%*	
Drug-related	*4*	*8.8%*	
Ocular	4	8.8%	
Temporary inflammatory	4	8.8%	
Syndrome***	2	4.4%	
Systemic	1	2.2%	
Rash***	1	2.2%	
Pancytopenia***			

^a^Median (IQR)

*CI* Confidence interval, *IAC* Intra-arterial chemotherapy

^*^Categorized as Grade 3a, which required additional post-procedure therapy without post-procedure sequelae, according to the Modified CIRSE Classification System for Complications [17]

^**^Categorized as Grade 1a, which observed during the procedure and could be resolved within the same session and the intended procedure was completed; no additional post-procedural therapy, no post-procedure sequelae, no deviation from the normal post-procedural course, according to the Modified CIRSE Classification System for Complications [17]

^***^Categorized as Grade 2, which resulted resulted in a deviation from the normal post-therapeutic course, necessitating prolonged observation including an overnight stay (< 48 h), but required no additional post-procedure therapy and resulted in no permanent sequelae, according to the Modified CIRSE Classification System for Complications [17]

In Group 1, two eyes (33.3%) required no further treatment; the remainder underwent additional IVC (50%), cryotherapy (66.6%), or IvitC (33.3%). Group 2 eyes had previously received systemic and/or local therapies (Table 3).

**Table 3:** Treatment and follow-up data

	*n* =	%
Treatment group		
IAC as first line treatment	6	40%
Additional treatments after IAC		
None	2	33.3%
IVC	3	50%
Cryotherapy	4	66.6%
IvitC	2	33.3%
Enucleation	0	0%
IAC as globe salvage therapy	9	60%
Previous treatments before IAC		
IVC	9	100%
Cryotherapy	6	66.6%
TTT	1	11.1%
IvitC	1	11.1%
Follow-up (months)^a^	24.5 (17–31.5)	
minimum–maximum	*12–85*	

^a^Median (IQR)

*IAC* Intra-arterial chemotherapy, *IVC* İntravenous chemotherapy, IvitC İntravitreal chemotherapy, TTT Transpupillary thermotherapy

Median follow-up was 24.5 months (range, 12–85 months). Visual acuity was assessed by our experienced ophthalmology team, using either the Fix&Follow method (12 eyes) or the Snellen chart (three eyes), depending on the patient's age and level of cooperation. At baseline, most eyes showed impaired function; at follow-up, visual acuity improved in one eye (6.7%), remained stable in 13 (86.6%), and worsened in one (6.7%) (Table 4). Overall, visual function was preserved or improved in 93.3% of the treated eyes (14/15; 95% CI 68.1–99.8%). There was no statistically significant difference in visual acuity outcomes between Group 1 and Group 2 when comparing initial presentation to the final follow-up (*p* = 1.000).

**Table 4:** Clinical presentation and treatment outcomes of 15 eyes treated with IAC, including visual acuity scores, metastasis, enucleation status, and mortality

Patient number	AJCC stage	ICRB group	Treatment group	IAC Cycle	Presentatıon			Outcome				
					Visual acuity		Metastasis	Visual acuity		Metastasis	Enucleation	Mortality
					Fix & Follow	Snellen		Fix & Follow	Snellen			
7-R 7-L	1 1	C D	1	4 10	1 1	N/A N/A	0	1 1	N/A N/A	0	0 0	0
5	1	E	1	1	N/A	3	0	N/A	3	0	0	0
11	1	D	1	2	N/A	2	0	N/A	2	0	0	0
12-R 12-L	1	D D	1	2 2	0 0	N/A N/A	0	0 0	N/A N/A	0	0 0	0
1	1	B	2	6	2	N/A	0	2	N/A	0	0	0
2	1	D	2	6	0	N/A	0	1	N/A	0	0	0
3	1	E	2	11	N/A	3	0	N/A	3	0	0	0
4	3	D	2	7	2	N/A	0	1	N/A	0	0	0
6	1	D	2	8	2	N/A	0	2	N/A	0	0	0
8	1	D	2	6	2	N/A	0	2	N/A	0	0	0
9	1	D	2	6	0	N/A	0	0	N/A	0	0	0
10	1	C	2	6	1	N/A	0	1	N/A	0	0	0
13	1	D	2	6	0	N/A	0	0	N/A	0	0	0

Each patient was evaluated using a single visual acuity testing modality at both the initial presentation and the final follow-up visit

Fix&Follow scores were defined as follows: 0 = fixation and following, 1 = only fixation, and 2 = no fixation and no following

The Snellen chart scores were categorized as follows: 1 = visual acuity better than 6/12; 2 = visual acuity between 6/12 and 6/60; and 3 = visual acuity ranging from 6/60 to light perception

*AJCC* American Joint Committee on Cancer, *ICRB* International Classification of Retinoblastoma, *N/A* not applicable

In this cohort, the event-free ocular survival rate was 100% (95% CI 78.2–100.0%) in both groups (*p* = 1.000). During this limited observation period, no instances of enucleation, metastasis, or mortality were recorded (Table 4).

## Discussion

This single-center retrospective study demonstrates the feasibility, safety, and efficacy of IAC in the management of RB, both as a first-line and a salvage treatment. Our findings align with a growing body of evidence supporting IAC as a globe-sparing modality capable of delivering high intraocular drug concentrations while minimizing systemic toxicity.

Although both groups achieved excellent globe salvage rates, the salvage therapy group required a significantly higher number of treatment cycles (*p* = 0.005) to achieve these outcomes. This observation suggests that while the triple-drug protocol is effective in eyes that have failed prior therapies, a more intensive treatment regimen may be necessary in the salvage setting than in treatment-naive cases.

In our cohort, the technical success rate was 97.8%, with superselective OA catheterization achieved in 44 of 45 sessions. One failed attempt due to vasospasm and OA tortuosity was successfully repeated after two weeks. Despite high feasibility, vascular anatomical challenges are well documented [11]. Gobin et al. reported a 98.5% success rate, with difficulties linked to ICA tortuosity, MMA inaccessibility, and limitations of the Japanese technique in younger patients [7]. Stenzel et al. described technical issues in 42% of cases, including catheter instability and orbital supply variations [19]. While the OA usually originates from the ICA, variants from the MMA or posterior circulation have been reported in 1.4–2.5% of cases [20–22]. We observed a meningo-ophthalmic variant in four patients, one of whom developed transient hyperemia without necrosis. Accessory MMA branches arising from the OA were also noted in two cases.

Concerns about IAC safety persist, especially regarding systemic and intra-procedural risks. In our cohort, no major complications occurred. Transient bronchospasm (6.6%) and hypotension (2.2%) were managed promptly; bronchospasm cases required temporary intubation. Similar complications, including superficial femoral artery occlusion and contrast reactions, were reported by Gobin et al. [7]. While severe events such as stroke, dissection, or death have been described [2, 11, 23, 24], none occurred in our series. Ocular complications were mild, mainly periocular inflammation (8.8%), and resolved without sequelae. Although rare, severe adverse events such as vascular occlusions or retinal detachment have been reported, potentially associated with rapid tumor regression and subsequent vascular instability [2, 11]. Systemic toxicity was limited to transient rash and pancytopenia (4.4%), managed conservatively. Hematologic profiles were favorable compared to prior studies: neutropenia rates up to 11.4% were reported at higher melphalan doses [7], while pooled analyses showed 5.9% neutropenia and 0.2% transfusion rates [11, 24]. These findings highlight the safety of IAC when integrated into tailored, multidisciplinary care pathways.

Our primary outcomes regarding event-free ocular survival, metastasis, and mortality reflect the safety and efficacy of the triple-drug IAC protocol within our study population. During a median follow-up of 24.5 months, no enucleations, metastases, or deaths were recorded, resulting in a 100% event-free ocular survival across both groups. Our findings remain encouraging for the short- to mid-term safety of the IAC procedure. In another comparative study of unilateral group D disease, IAC achieved 100% globe salvage at 41.7 months, compared to a 43% enucleation rate with systemic chemotherapy [17]. While these results align with the upper ranges of success reported in the literature, they must be interpreted with caution and require longer-term data to determine whether they remain stable over time. For instance, Gobin et al. reported a 2-year survival of 70% overall (81.7% for primary IAC and 58.4% for salvage cases), with lower rates in Reese-Ellsworth group V eyes [7]. Although our 100% survival rate appears favorable in comparison, the inherent differences in sample size, follow-up duration, and patient heterogeneity limit the direct comparability of these cohorts. Other series have reported varying salvage rates, such as 100% in groups B/C but significantly lower rates in group E, which are often attributed to differences in cohort composition [24]. While the majority of eyes in our cohort were classified as ICRB group D and E, the one-year event-free ocular survival remained 100% across all groups. This favorable outcome may also be attributed to our comprehensive treatment approach; specifically, all group E eyes in our cohort received IvitC following IAC, which played a critical role in improving tumor control and avoiding enucleation. Munier et al. [17] also highlighted IvitC as an effective consolidation therapy while reinforcing the intrinsic efficacy of IAC compared to systemic chemotherapy-based strategies. In the salvage group, the cumulative effect of prior systemic chemotherapy may have also contributed to the favorable outcomes by reducing initial tumor volume or priming the intraocular environment for subsequent IAC. This synergistic application of localized and systemic modalities, with careful case selection, underscores the importance of a comprehensive treatment strategy to preserve the globe in high-risk eyes. Nonetheless, long-term follow-up remains essential to assess whether these early favorable outcomes persist, particularly in patients with high-risk features such as vitreous seeding.

This study has several limitations, including its retrospective, single-center design, small sample size, and absence of a control group, all of which limit generalizability. Heterogeneity in prior treatments among salvage cases may also introduce bias. Although 12 months of follow-up is clinically meaningful and we achieved 100% globe salvage and event-free survival, these findings should be contextualized within the study’s limitations. A longer observation period is required to evaluate delayed complications and recurrence. In addition, potential selection bias should be considered, as patients referred to our center may have already been deemed suitable for globe-sparing therapy, which could limit the generalizability of the findings. Future prospective multicenter studies with larger and more diverse cohorts are needed. Despite these limitations, our results provide valuable real-world data from a specialized IAC center.

## Conclusion

Our single-center experience suggests that IAC is a technically feasible and promising option for both primary and salvage treatments of intraocular RB. In our cohort, the procedure achieved high technical success with minor, manageable complications and minimal systemic toxicity. During a median follow-up of 24.5 months, preliminary outcomes showed preserved visual function and high ocular survival, with no metastasis or mortality. While these findings are encouraging, further large-scale studies with longer follow-up are warranted to confirm the long-term efficacy and safety of IAC as a globe-sparing strategy within a multidisciplinary framework.

## Data Availability

The datasets generated and/or analyzed during the current study are available from the corresponding author upon reasonable request.
